# The Equatoguinean Malaria Vaccine Initiative: From the Launching of a Clinical Research Platform to Malaria Elimination Planning in Central West Africa

**DOI:** 10.4269/ajtmh.19-0966

**Published:** 2020-05-26

**Authors:** Peter F. Billingsley, Carl D. Maas, Ally Olotu, Christopher Schwabe, Guillermo A. García, Matilde Riloha Rivas, Dianna E. B. Hergott, Claudia Daubenberger, Elizabeth Saverino, Adel Chaouch, Oscar Embon, Mwajuma Chemba, Elizabeth Nyakarungu, Ali Hamad, Carlos Cortes, Tobias Schindler, Maximillian Mpina, Ali Mtoro, B. Kim Lee Sim, Thomas L. Richie, Ken McGhee, Marcel Tanner, Gabriel Mbaga Obiang Lima, Salim Abdulla, Stephen L. Hoffman, Mitoha Ondo’o Ayekaba

**Affiliations:** 1 Sanaria Inc., Rockville, Maryland;; 2 Marathon Oil, Malabo Dos, Bioko Norte, Equatorial Guinea;; 3 Ifakara Health Institute, Bagamoyo, Tanzania;; 4 KEMRI Wellcome Trust Research Programme, Kilifi, Kenya;; 5 Medical Care Development International, Silver Spring, Maryland;; 6 Ministry of Health and Social Welfare, Government of Equatorial Guinea, Malabo, Equatorial Guinea;; 7 Swiss Tropical and Public Health Institute, Basel, Switzerland;; 8 University of Basel, Basel, Switzerland;; 9 La Paz Hospital Medical Center, Sipopo, Equatorial Guinea;; 10 Noble Energy, Malabo Dos, Equatorial Guinea;; 11 Ministry of Mines and Hydrocarbons, Government of Equatorial Guinea, Malabo Dos, Equatorial Guinea

## Abstract

Fifteen years of investment in malaria control on Bioko Island, Equatorial Guinea (EG), dramatically reduced malaria-associated morbidity and mortality, but the impact has plateaued. To progress toward elimination, EG is investing in the development of a malaria vaccine. We assessed the unique public–private partnership that has had such a significant impact on malaria on Bioko Island and now added a major effort on malaria vaccine development. As part of a $79M commitment, the EG government (75%) and three American energy companies (25%) have invested since 2012 greater than $55M in the Equatoguinean Malaria Vaccine Initiative (EGMVI) to support clinical development of Sanaria^®^ PfSPZ vaccines (Sanaria Inc., Rockville, MD). In turn, the vaccine development program is building human capital and physical capacity. The EGMVI established regulatory and ethical oversight to ensure compliance with the International Conference on Harmonization and Good Clinical Practices for the first importation of investigational product, ethical approval, and conduct of a clinical trial in Equatoguinean history. The EGMVI has completed three vaccine trials in EG, two vaccine trials in Tanzania, and a malaria incidence study, and initiated preparations for a 2,100-volunteer clinical trial. Personnel are training for advanced degrees abroad and have been trained in Good Clinical Practices and protocol-specific methods. A new facility has established the foundation for a national research institute. Biomedical research and development within this visionary, ambitious public–private partnership is fostering major improvements in EG. The EGMVI plans to use a PfSPZ Vaccine alongside standard malaria control interventions to eliminate Pf malaria from Bioko, becoming a potential model for elimination campaigns elsewhere.

## INTRODUCTION

Recently, African heads of states endorsed the African Union Agenda 2063, calling for investments in science, technology, research, and innovation to build prosperous and sustainable economies,^1–3^ resulting in some countries investing in biomedical research addressing local pressing health issues^4,5^ This article describes work done through investments made by the government of Equatorial Guinea (GEG) in malaria vaccine development and the GEG’s interest in deploying Sanaria’s *Plasmodium falciparum* sporozoite (PfSPZ) Vaccine to eliminate *Plasmodium falciparum* (Pf) malaria from selected regions. Equatorial Guinea (EG) is an oil and gas–producing Central African country divided into mainland and insular regions. Bioko (population ∼280,000) is the most populous island and the location of the country’s capital, Malabo.^6^ Since discovery of offshore oil in the 1990s, EG has boomed economically,^3^ with significant improvements in public health exemplified by the building of modern hospitals such as the La Paz Medical Center.^5,7–10^ However, malaria remains a major public health problem in Africa,^11,12^ including in EG where there is markedly higher prevalence on the mainland and other islands of EG, accounting for ∼50% of illness in children younger than 5 years^6,10^ and significantly impacting the Equatoguinean economy.^7,13,14^

### Intractable malaria.

**Figure 1.**
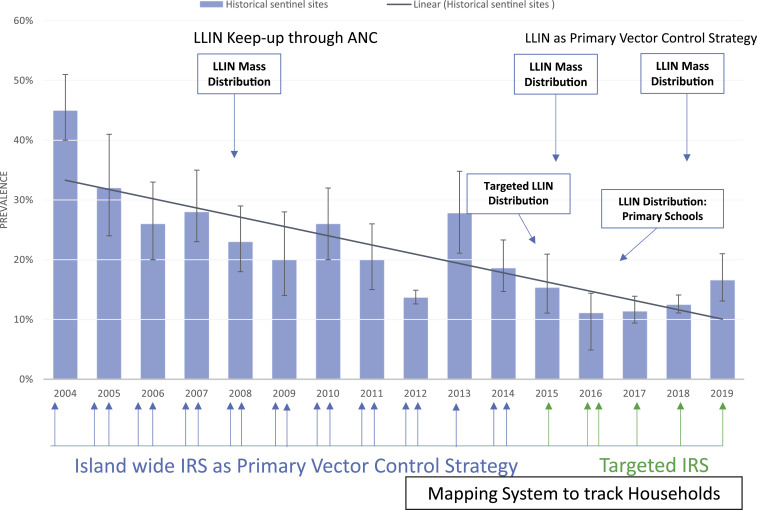
Prevalence of *Plasmodium falciparum* in 2- to 14-year-old children and historical vector control interventions on Bioko Island. With a combination of indoor residual spraying with insecticides, coverage with long-lasting insecticide-treated nets (LLINs), and improved case management, malaria prevalence measured by the same rapid diagnostic test declined from 45% in 2004 to 13.7% in 2012 but only to 16.6% in 2019. The implementation has been supported by a growing database composed of a mapping system that helps identify households that will be sprayed and/or receive LLINs. ANC = antenatal care program. This figure appears in color at www.ajtmh.org.

In 2018, malaria caused an estimated 228 million clinical episodes and 405,000 deaths worldwide.^12^ In Africa, the economic impact of malaria is estimated to be 5–6% of the nations’ gross domestic product.^12,15^ Despite an annual international investment in malaria control of greater than $4.3 billion,^16^ annual malaria cases and deaths were effectively unchanged from 2015 to 2017.^12^ The impact of malaria control on Bioko Island parallels these global trends. Historical data from annual malaria indicator surveys in sentinel sites conducted by the Bioko Island Malaria Control Project (BIMCP) show an appreciable drop in malaria prevalence in 2- to 14-year-old children from 2004 (45%) to 2012 (14%) (Figure 1), followed by a plateau through 2018 and a slight increase in 2019 (16.6%).^5–7^ Current tools alone, even when applied in an integrated and progressively targeted manner over 15 years, appear to be insufficient to halt malaria transmission on Bioko, which is augmented by continuous importation of malaria parasites from the mainland.^5,14,17^ A vaccine that prevents infection in individuals and has a “mass effect,” often referred to as herd immunity, in the community would be an ideal tool to acheive malaria elimination when used in conjunction with other control measures. PfSPZ vaccines have a proven record of preventing Pf infections in controlled human malaria infection (CHMI) studies and natural transmission settings, and are among the most advanced malaria vaccine candidates.^18–26^ PfSPZ Vaccine has been selected as the first-generation candidate to develop for inclusion in future malaria control and elimination programs in EG.^27^

### The BIMCP.

The BIMCP was launched in 2004 as a public–private partnership to control and eventually eliminate malaria on Bioko. The BIMCP was funded by the GEG and private partners: Marathon Oil, Noble Energy, and Atlantic Methanol Production Company,^7,28,29^ implemented by a U.S. nongovernmental organization, Medical Care Development International (MCDI), in collaboration with the Equatoguinean National Malaria Control Program within the Ministry of Health and Social Welfare (MHSW). The project deployed numerous malaria control interventions, including indoor residual spraying of insecticides, achieving at least 80% of targeted coverage during island-wide and targeted spray rounds, mass distribution and installation of long-lasting insecticide-treated nets, achieving at least 85% coverage during each distribution campaign, coupled with targeted maintenance through antenatal clinics and schools, focal application of larvicides, universal curative treatment with artemisinin combination therapy, intermittent preventive treatment in pregnancy, mass education and behavioral change communications, and training MHSW personnel.^5,9,13,30^ In addition, the BIMCP established a mapping and enumeration system of houses and households that allowed it to more effectively plan, implement, target, and monitor malaria control interventions. The implementation of this system began in 2012 and was fully implemented in 2014. As a result, malaria prevalence in 2- to 14-year-old children living on Bioko was reduced by 63% from 2004 to 2019^5,31^ (Figure 1), and there has been a 64% reduction in child mortality (from 152 to 55 deaths per 1,000 births) since inception.^5,7^ Since 2015, when sampling of households for the annual malaria indicator survey expanded to all communities on Bioko, the overall island-wide all age prevalence of malaria parasites has decreased from 12.7% (2015)^14,32^ to 10.3% (2018).^33^ However, evidence indicates that the odds of malaria infection in travelers to mainland EG are between 3 and 4 times higher than for individuals who do not travel, indicating that the persistent levels in prevalence are in part attributable to importation by travelers between Bioko and the mainland.^5,14,17^

### *Plasmodium falciparum* sporozoite vaccines.

Sanaria Inc. has developed a platform technology for producing aseptic, purified, cryopreserved PfSPZ in compliance with Good Manufacturing Practices.^19^ Sanaria’s product portfolio includes Sanaria^®^ PfSPZ Vaccine (radiation-attenuated PfSPZ),^18,20–24,27^ PfSPZ Challenge which is composed of infectious PfSPZ used for CHMI,^25,34–42^ and PfSPZ-GA1 (genetically attenuated PfSPZ).^19^ PfSPZ-CVac (chemo-attenuated PfSPZ) combines PfSPZ Challenge with antimalarial drugs.^19,43–45^ In clinical trials in six countries in Africa, two countries in Europe, and five sites in the United States, PfSPZ-based vaccines have been consistently safe and well tolerated. They have protected greater than 90% of recipients against CHMI in clinical trials conducted in the United States, Germany, Tanzania, and Mali^24,26,43,46^ (Sissoko, unpublished), with protection lasting for at least 8 months against heterologous CHMI^20^ and 14 months against homologous CHMI.^22^ Protection of approximately 50% lasting for at least 6 months and in one case at least 18 months against naturally transmitted malaria has been demonstrated in four independent clinical trials in Mali^24^ and Burkina Faso (Sissoko, unpublished; Sirima, unpublished).

## APPROACH AND DISCUSSION

### The Equatoguinean Malaria Vaccine Initiative (EGMVI).

In 2012, Sanaria and the Ifakara Health Institute (IHI, Tanzania) leadership met with the GEG, and Marathon Oil to collaborate with MCDI to establish the EGMVI and test Sanaria^®^ PfSPZ Vaccine on Bioko Island. The decisions to move forward in this unique partnership were based on a recognition that current control approaches had reached maximum capacity and that an efficacious and effective vaccine to prevent infection and transmission would be an ideal tool to reach elimination on Bioko. The absence of any such licensed vaccine attracted the funders to support development of PfSPZ Vaccine and the desire of the GEG to become part of a vaccine development and clinical research effort also drove the stepwise decision-making through the initiation and subsequent ramping up of the EGMVI. After a contribution by the BIMCP funding partners of $3.8M in 2012 to initiate the first clinical trial, the EGMVI was funded in 2015 with an additional $44M, 75% of the funding coming from the GEG. MCDI became responsible for management, partner and government relations, recruitment and deployment of staff, logistics, and procurement. The Swiss Tropical Public Health Institute (Swiss TPH) provided laboratory support and assistance in strategy and design, and IHI, in a novel South–South collaboration, was engaged for providing personnel and training to launch the EGMVI clinical trial program and establish an institutional mechanism for sustainability of research resources. Because there had been no prior national experience with clinical research compliant with international standards, several major tasks were set before starting a trial:

1. establish understanding of clinical trial processes, compliance, ethical requirements, and research procedures at all levels of government and in the general population;
2. build human resource capital and clinical trial infrastructure;
3. establish a national ethical review committee;
4. establish regulatory capacity to import investigational product; and
5. establish institutional framework and capacity within the MHSW to meet its legal and regulatory mandate of sustaining the research resources of the trials.

The EGMVI recruited a team of African, European, and American experts in malaria to form a scientific advisory group.† EGMVI partners began engaging with the MHSW and other governmental organizations like the National University of EG, the Ministry of Education and Science, parliament, and the Presidency, giving appropriate informational presentations. The EGMVI laid out the requirements for clinical research and defined the unique role for EG in the development of PfSPZ vaccines and their deployment as public health tools. Defining the partnership and training was important in these early discussions. The IHI provided opportunities to visit their research facility in Bagamoyo and trained GEG officials on what EGMVI would require to be successful. Excellent international-standard medical services were made available to host the first vaccine trial in EG. To further expand training opportunities for EG personnel and pre-test all procedures for vaccine studies, the EGMVI committed to run studies at the established IHI site in Bagamoyo, Tanzania. Hence, the first trial in EG (EGSPZV1) confirmed the tolerability, safety, and immunogenicity of PfSPZ Vaccine in 33 male (ages 18–35 years) subjects, similar to a prior study in Bagamoyo (BSPZV1).^27^ Subsequently, an age de-escalation/escalation trial (EGSPZV2) followed the initiation of a parallel trial (BSPZV2) in Tanzania also EGMVI-funded.^47^. As part of the commitment, the GEG funded the EGMVI team to conduct a critical assessment of PfSPZ Vaccine in HIV-infected subjects in Bagamoyo (BSPZV3). To our knowledge, this is the first time that an African state funded a portfolio of clinical research conducted in a partner African state, in large part because it funded the EGMVI as a learning and partnership activity. The partnership is also unique in supporting industry R&D needed for a commercial product.

### Establishing human capital and infrastructure capacity.

Initiation of the EGSPZV1 study in EG was challenging. Given the lack of experience, and with volunteer safety being paramount, clinical, administrative, laboratory, and training teams were established in which Equatoguineans were embedded into the clinical (IHI), laboratory (Swiss TPH), and hospital (La Paz Hospital, Sipopo) teams to ensure proper training and supervision. MCDI managed logistics to execute the trials, including working closely with the MHSW to recruit Equatoguinean team members who were seconded from the MHSW to the EGMVI. The IHI provided expatriate staff and training for Equatoguinean nationals, as well as ethical oversight. Swiss TPH provided ethical oversight, and organized, equipped, and ran the trial laboratories. La Paz Hospital, managed by an Israeli team, provided staff and access to hospital space for subject evaluation, vaccine administration, and laboratory evaluation. Sanaria, Inc. (Rockville, MD), as project sponsor, provided clinical support and worked with the La Paz pharmacy to receive and prepare vaccine in collaboration with an Equatoguinean pharmacist. Marathon Oil and its partners, led by Marathon’s Corporate Social Responsibility personnel based in EG, acted as a liaison to the highest level within the GEG to inform the GEG about progress and challenges. When it became necessary to expand the trial team for future trials and hire non-MHSW national staff, a human resource and training committee consisting of members of the MHSW, MCDI, Sanaria, and Marathon Oil staff was created to assure transparency and legitimacy in the staff selection process. This process facilitated the hiring of critical non-MHSW national staff after the first trial.

In addition, the Equatoguinean staff received hands on training from the Malaria Research and Training Center (University of Bamako, Mali) in Donéguébougou, Mali, during a clinical trial of PfSPZ Vaccine.^24^ A NIH training consultant and Sanaria staff helped develop a data management framework and an approach for quality systems review. For Equatoguineans, it was both inspiring and challenging to work with new colleagues from various countries with a broad array of professional experience, providing them with many opportunities to improve their clinical, analytical, and research skills.

Clinical laboratories processing specimens from clinical trials require an appropriate set of standards to guide good practices.^48^ The EGMVI in collaboration with La Paz Hospital worked to improve the quality of the hospital clinical laboratory. Initially, the IHI and La Paz laboratory managers, with support from Swiss TPH and Sanaria, worked together to implement a four-stage quality management system that complied with Good Clinical Practices and ISO 15189. This effort led to the La Paz laboratory being considered a three-star–rated laboratory based on the WHO-AFRO-SLIPTA assessment, thereby allowing the laboratory to serve the trial. In recognition of the need for a dedicated research laboratory to support subsequent clinical trials, GEG and private partners funded the construction of a state-of-art laboratory and clinical center in Baney city in Baney district (a community approximately 25 km southeast of Malabo), which was completed and inaugurated in 2019. With scientific oversight from Swiss TPH and the IHI, the new laboratory will conduct safety, molecular, and immunological analyses of clinical trial samples to determine safety and mechanism of action of PfSPZ Vaccine. The laboratory will facilitate the transfer of technology from more advanced laboratories in Europe and the United States to EG and build local expertise in biomedical sciences that will support other diseases studies and address relevant research questions. For example, on 30 January 2020, the Baney laboratory, built for malaria trials, conducted the first SARS-CoV-2 qPCR in Equatorial Guinea, and possibly in Africa. The clinical section of the building will support the clinical care of patients as Baney District Hospital.

### Establishing ethical and regulatory structures.

Before the EGMVI, there was no independent national ethics committee in EG. Observational studies, cluster-randomized trials,^49^ and surveys had received ethical approval from *ad hoc* ethics committees within the MHSW or participating institutions.^49,50^ A national, independent ethics committee (Comité Ética Nacional de Guinea Ecuatorial—CENGE) was established in October 2014 to review and approve human research protocols in the country, starting with the first clinical trial, EGSPZV1. The IHI, WHO-AFRO, and the University of Maryland provided specific technical suggestions about the structure and processes of CENGE, and the EG MHSW selected the initial committee members. The CENGE was trained by experts in human research ethics from WHO-AFRO, IHI Institutional Review Board (IRB), Tanzanian National Health Research Ethics Committee, and the University of Maryland, Baltimore IRB chair with ongoing support from MCDI. The IHI IRB shared its procedural template with CENGE, and the WHO-AFRO invited and integrated the leadership of CENGE into the African Vaccine Regulatory Forum (AVAREF), with the aim of providing additional technical support to improve CENGE’s capacity and efficiency.

The EGMVI also trained the Department of Pharmacy and Traditional Medicine (DPTM) (MHSW) staff, which is responsible for reviewing all drug registration and importation into the country. Before EGMVI, the DPTM lacked the capacity and knowledge to review investigational products for clinical trials. The DPTM received training on regulations related to clinical trial reviews and the roles and responsibilities of regulatory authorities in studies involving human subjects. The EGMVI provided the DPTM with policies and procedures for importation of investigational product used by ministries of health of Mali, Ghana, and Tanzania, ensuring that the director had both technical knowledge and reassurance that these procedures are used by other African states. The DPTM director and Sanaria’s chief scientific officer attended an AVAREF meeting in 2013, which provided further opportunities to interact with other African counterparts.

### Conducting the first vaccine trials in EG.

Results of the first clinical trial, EGSPZV1, in EG were published in 2018.^27^ Planning and protocol development began in mid-2014, and volunteer screening started in March 2015. The first step involved high-level meetings with the president of EG, the Minister of Mines and Hydrocarbons, and two successive ministers of Health and Social Welfare to provide a detailed understanding of the project. Subsequent meetings with the MHSW reviewed the community engagement plan and the study protocol. The EGMVI team engaged the Ministry of Interior and Ministry of Education and Science, where plans were further reviewed, discussed, and approved. The Ministry of Interior provided the necessary approvals allowing political leadership in the study area to review the recruitment plans and processes. In addition, the EGMVI team delivered an in-depth presentation to the Equatoguinean parliament, creating the opportunity for the members of parliament to ask questions regarding the study and long-term project goals.

A community engagement plan was then developed. MCDI recruited a U.S.-trained, Equatoguinean public health professional who was a member of the primary ethnic group of the study area and spoke the local language, and who could therefore effectively engage both the international staff and the community to build trust in and affinity with the project. Community meetings were held with village presidents in the study area, followed by prescreening meetings, and finally screening visits where volunteers were consented and assessed for the eligibility criteria. After the studies were completed, meetings were held with target communities to report study findings. Based on the lessons learned (Box 1) and clinical trial results, EGMVI is planning the first clinical trial of phase 3 compliant PfSPZ Vaccine, a trial which will provide data to support the application for licensure of PfSPZ Vaccine.

### Long-term human resource capital building.

Another critical component of the capacity-building exercise has been providing opportunities for Equatoguineans to obtain higher degrees. Through EGMVI funding, Equatoguinean team members were offered the opportunity to identify courses and programs for self-development, with support from MCDI in the application process. To date, six Equatoguinean nationals have enrolled for higher degrees in Spain (IS Global), the Netherlands (the University of Utrecht), the United Kingdom (the University of Manchester), and Switzerland (the University of Basel). Each has a research element to their degree that will contribute to the EGMVI goals and supported on the ground by experienced IHI, Swiss, and Sanaria supervisors. Other staff have enrolled in online certifications and diplomas. These self-motivated, internationally educated staff are critical to the long-term stability, growth and success of the EGMVI and the sustainability of research resources through a national public health institute, the Instituto Nacional de Investigación en Salud Pública de Guinea Ecuatorial (INISAPGE).

### Instituto Nacional de Investigación en Salud Pública de Guinea Ecuatorial.

Previously, the MHSW had no mechanism or processes to coordinate and manage clinical research in EG. Furthermore, implementation of EGMVI trials generated data and biological sample resources that required MHSW maintenance in compliance with international legal and regulatory standards. Within the implementation of EGMVI, the IHI led the establishment of such a framework for the MHSW in an exemplary South–South cooperation approach. A joint IHI and MHSW team developed the articles of establishment for INISAPGE. Administrative manuals and procedures of the institute were presented and endorsed by various government officials and health stakeholders. Instituto Nacional de Investigación en Salud Pública de Guinea Ecuatorial was established in February 2019 to be the research arm of the MHSW, coordinating all human health research conducted in EG, as well as facilitating the development and implementation of an agenda promoting rapid health transformation in the country. Additional research projects are developing in partnership with the WHO and academic and private institutions, as well as hosting students including a PhD candidate supported by OCEAC (the agence d’exécution de la CEMAC (Central African Economic and Monetary Community)). INISAPGE will inherit the responsibility for maintaining clinical research resources in EG and be the host institution for the trained EGMVI scientific staff.

### FUTURE PLANS AND PERSPECTIVE

**Figure 2.**
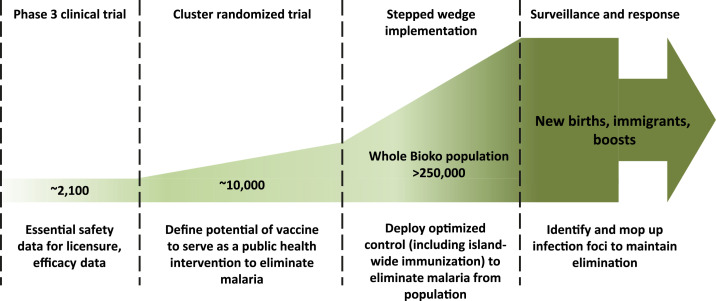
Proposed pathway to the elimination of *Plasmodium falciparum* malaria on Bioko Island. This figure appears in color at www.ajtmh.org.

In 2021, the Bioko Island Malaria Elimination Program (BIMEP), the merger of BIMCP and EGMVI, will launch a clinical trial with phase 3 compliant PfSPZ Vaccine. A licensed PfSPZ Vaccine is critical to the long-term plans for malaria control and elimination (Figure 2), and this clinical trial has the explicit aim of obtaining data for licensing Sanaria^®^ PfSPZ Vaccine in the United States, Europe, and at least one African country with well-established regulatory procedures. There will be two parts to this clinical trial, both intended to develop systems, facilities, and personnel at the next level of research, compliance, and responsibility. Two thousand one hundred Equatoguinean volunteers aged 2–50 years will receive PfSPZ Vaccine or placebo, be boosted 9 months later, and then followed up to demonstrate safety, immunogenicity, and protective efficacy. The ambitious goal is to have a regulatory submission and licensed product by 2022/23, which may be possible given that Sanaria has been granted FDA “Fast Track” status for PfSPZ Vaccine.

With a trained clinical research workforce, infrastructure, and institutional framework in place, once the product is licensed, the BIMEP aims to demonstrate the public health utility of PfSPZ Vaccine within a fully integrated malaria control program. With individual protective efficacy demonstrated (Figure 2), a cluster-randomized clinical trial will then evaluate PfSPZ Vaccine for a possible vaccine mass effect or herd immunity, and interruption of malaria transmission within the vaccine-treated clusters. The plans for this demonstration project include modeling to define matching paired clusters of similar malaria epidemiologies and population demographics across Bioko Island. The BIMEP will then seek to move into an elimination program based on a stepped-wedge design (Figure 2). Here, established malaria control will be combined with mass drug administration and mass vaccination programs across the whole island to achieve Pf elimination. The program will additionally have two important complementary components: 1) curative use of drugs and prophylactic (vaccine) treatments of travelers to and from the mainland to reduce the probability of infection when on the mainland and reintroductions when returning to Bioko, and 2) a surveillance-response system based on rapid diagnosis combined with response packages that also include vaccination. A georeferenced demographics database will identify new people needing vaccinations (e.g., children as they reach 2 years of age, immigrants). This will be a comprehensive but massive undertaking, targeting more than 280,000 people for full coverage. However, if Pf can be eliminated at this scale from a region that once had one of the highest transmission intensities (entomological inoculation rates) in Africa,^51,52^ then the stage will be set for an external validation of this approach, leading through generalization to comparable implementation throughout diverse African malaria epidemiological settings to halt the transmission and eliminate Pf malaria systematically from defined geographic areas in Africa and eventually the world.

Box 1 **Lessons learned from establishing the EGMVI**

Based on feedback from the funders, project partners, project staff, consultants, and outside experts who participated in annual meetings to review the project’s planning and progress, the following lessons learned were taken from the experience:

A broad partnership approach across the public, private, and NGO sectors is key, and large time frames are required to work in such an inexperienced, scientifically immature environment. Typically, double or triple the amount of time was required to complete tasks compared with in established clinical research environments. Generous planning and scheduling recommended to avoid unplanned costs and milestone planning must be conditioned to account for unexpected delays. Controlling internal factors, such as staff performance expectations, should be a major management focus to hold research team and leadership accountable.

Early and continuing, iterative consultative meetings were important in creating awareness of the EGMVI plans and later the actual work, and generating support for the clinical trial work. This applied to local communities, the CENGE, and executive- and congressional-level government.

Must consider the political context of the country when planning a research program. Whereas in some countries a bottom-up approach might be more effective, in EG, a top-down approach was central to the project’s success, as granting political permission was needed to ensure support of lower level administrators and bureaucrats.

Organigrams that clearly identify roles and responsibilities for the project at all levels needed to be developed early, adhered to, and updated regularly based on performance so that activities are not allocated to an individual who is not suited or able to execute the planned tasks.

Funding agreements should be set in 3- to 5-year increments with opportunities to review progress. To control costs, budgets should be reviewed and approved annually, and cost-tracking measures/schemes must be in place. This ensures that there is a long-term commitment, while also ensuring accountability.

Capacity building takes enormous effort and should be approached with a broad perspective. For instance, based on our partnership approach, the local staff were included in international conferences and meetings, with the expectation that they will actively participate at the same level as other contributors, giving talks and presenting data. Hence, the prerequisite for local (and expat) staff was to have an accepted posters and/or oral presentations to attend meetings and conferences. This created an internal meritocracy that supported effort and precluded political influences and preferences as to who represents the project internationally.

Include GEG officials in annual planning and review meetings. This is important so that there is governmental oversight and, importantly, a safe place for GEG officials to ask questions. Government officials were also invited to international partner meetings so that GEG officials could see and hear about other trials being conducted on *Plasmodium falciparum* sporozoite Vaccine, their findings, and the challenges. GEG officials could confirm from research teams in other countries that the product was being used elsewhere and there were no safety issues. Importantly, such interactions allowed for local scientific staff and GEG officials to hear the same information in real time, reducing the politicization of the information provided.

Government co-funding. For projects of such magnitude to be sustainable, there must be long-term financial support for the project. To create funding stability over time, an agreement between the private parties and the Ministry of Mines and Hydrocarbons was established that allowed credits to be applied toward the project that offset taxes. This allowed for the funding to be established and maintained over time, while also ensuring that the GEG had control over the funding, through annual letters of approvals signed by the Ministry of Mines and Hydrocarbons after annual budget and technical reviews.

The South–South relationships that emerged from the establishment of the EGMVI, as well as through the BIMCP, came about through two main activities: employing Southern vendors, like the Tanzanian IHI, and employing African and South American professionals. The vendors were needed because of their existing level of expertise, and the professional staff hired were needed to serve as role models to the local staff hired into the project. Both strategies are purposeful and required forethought and investment to make them work.

Effective governance, as noted in the text, is established by having routine, high-level meetings with ministerial and executive-level branches of government, as well as holding regular technical and financial meetings with mid- and upper-level GEG representatives, that ensure that there is understanding and buy-in for the investments being made and evidence of progress as well as setbacks that can be discussed and acted on. On the private vendor side, Marathon Oil held vendors accountable using standard contract and finance/accounting compliance tools. This exercise ensured that the GEG received up-to-date compliance data, as well as the private parties, ensuring continued trust required to commit to long-term funding over time. With respect to driving this governance, Marathon Oil CSR staff were responsible to maintain the viability of the CSR investment and hence drove the processes through direct engagement with the GEG via the Ministry of Health and Social Welfare, as well as through established annual production review meetings with the company and the Ministry of Mines and Hydrocarbons.

Strategies that worked best for the BIMCP/EGMVI program included creating HR committees and other mechanisms that gave GEG entry into the decision and resource deployment process, while also ensuring that the scientific and financial stakeholders had seats at these tables. There must be a willingness on the part of the financial stakeholders to engage all partners where they are at in developmental terms. What did not work well was the employment of staff who were directed by political actors such as ministers. This was not due to conflicts of interest, rather it was due to limited understanding of the needed skills and talents for a novel project in a limited human capacity environment.

## Footnotes

† Pedro L. Alonso, Fred. N. Binka, Brian M. Greenwood, Matthew B. Laurens, Seth Owusi-Agyei, Christopher V. Plowe, Thomas L. Richie, Robert W. Sauerwein, Sodiomon B. Sirima, Laurence Slutsker, Marcel Tanner, Peter Kremsner, Ogobara Doumbo.
